# Directly Sequenced Genomes of Contemporary Strains of Syphilis Reveal Recombination-Driven Diversity in Genes Encoding Predicted Surface-Exposed Antigens

**DOI:** 10.3389/fmicb.2019.01691

**Published:** 2019-07-31

**Authors:** Linda Grillová, Jan Oppelt, Lenka Mikalová, Markéta Nováková, Lorenzo Giacani, Anežka Niesnerová, Angel A. Noda, Ariel E. Mechaly, Petra Pospíšilová, Darina Čejková, Philippe A. Grange, Nicolas Dupin, Radim Strnadel, Marcus Chen, Ian Denham, Natasha Arora, Mathieu Picardeau, Christopher Weston, R. Allyn Forsyth, David Šmajs

**Affiliations:** ^1^Department of Biology, Faculty of Medicine, Masaryk University, Brno, Czechia; ^2^Biology of Spirochetes Unit, Institut Pasteur, Paris, France; ^3^CEITEC-Central European Institute of Technology, Masaryk University, Brno, Czechia; ^4^National Centre for Biomolecular Research, Faculty of Science, Masaryk University, Brno, Czechia; ^5^Department of Medicine, Division of Allergy and Infectious Diseases, University of Washington, Seattle, WA, United States; ^6^Department of Global Health, Harborview Medical Center, University of Washington, Seattle, WA, United States; ^7^Department of Mycology-Bacteriology, Instituto de Medicina Tropical “Pedro Kourí”, Havana, Cuba; ^8^Plateforme de Cristallographie, Institut Pasteur, Paris, France; ^9^Department of Immunology, Veterinary Research Institute, Brno, Czechia; ^10^Faculté de Médecine, Laboratoire de Dermatologie-CNR IST Bactériennes, Institut Cochin U1016, Université Sorbonne Paris Descartes, Paris, France; ^11^AP-HP, Service de Dermatologie et Vénéréologie, Groupe Hospitalier Paris Centre Cochin-Hôtel Dieu-Broca, Paris, France; ^12^Department of Dermatovenerology, University Hospital Brno, Brno, Czechia; ^13^Melbourne Sexual Health Centre, Alfred Health, Melbourne, VIC, Australia; ^14^Central Clinical School, Monash University, Melbourne, VIC, Australia; ^15^Zurich Institute of Forensic Medicine, University of Zurich, Zurich, Switzerland; ^16^GeneticPrime Dx, Inc., La Jolla, CA, United States; ^17^Department of Biology, San Diego State University, San Diego, CA, United States

**Keywords:** *Treponema pallidum* subsp. *pallidum*, syphilis, direct whole genome sequencing, recombination-driven diversity, culture-independent bacterial enrichment

## Abstract

Syphilis, caused by *Treponema pallidum* subsp. *pallidum* (TPA), remains an important public health problem with an increasing worldwide prevalence. Despite recent advances in *in vitro* cultivation, genetic variability of this pathogen during infection is poorly understood. Here, we present contemporary and geographically diverse complete treponemal genome sequences isolated directly from patients using a methyl-directed enrichment prior to sequencing. This approach reveals that approximately 50% of the genetic diversity found in TPA is driven by inter- and/or intra-strain recombination events, particularly in strains belonging to one of the defined genetic groups of syphilis treponemes: Nichols-like strains. Recombinant loci were found to encode putative outer-membrane proteins and the recombination variability was almost exclusively found in regions predicted to be at the host-pathogen interface. Genetic recombination has been considered to be a rare event in treponemes, yet our study unexpectedly showed that it occurs at a significant level and may have important impacts in the biology of this pathogen, especially as these events occur primarily in the outer membrane proteins. This study reveals the existence of strains with different repertoires of surface-exposed antigens circulating in the current human population, which should be taken into account during syphilis vaccine development.

## Introduction

*Treponema pallidum* subsp. *pallidum* (TPA) is the causative agent of syphilis, a globally occurring disease. Although the worldwide number of syphilis cases dramatically decreased after the introduction of penicillin therapy in the 1940s, the estimated number of new syphilis cases per year remains over 5.6 million. Especially alarming is the number of congenital syphilis cases, which is approaching one million cases per year (Newman et al., 2012; Peeling et al., 2017). In developed countries, syphilis is often transmitted among MSM patients (men who have sex with men). Moreover, MSM patients with syphilis are often co-infected with HIV (42% in Western Europe) (Dubourg et al., 2015). It is believed that syphilis facilitates the HIV infection, since syphilitic genital ulcers are infiltrated with lymphocytes (the primary target cells for HIV-infection) and provide a portal of entry for HIV acquisition. The rising prevalence of syphilis among MSM patients has coincided with the introduction of highly active anti-retroviral drugs leading to decreased HIV-associated mortality and the re-emergence of unsafe sexual behavior among MSM (Stolte et al., 2001). TPA infections are characterized by early and fast dissemination, immune evasion and long persistence in untreated patients. However, the underlying molecular mechanisms remain poorly understood (Radolf et al., 2016).

In spite of recent advances in *in vitro* cultivation of TPA (Edmondson et al., 2018), routine laboratory cultivation of this pathogen directly from patient samples is not yet possible. Therefore, most of the information on TPA genetics comes from genome sequencing studies, where DNA was isolated from bacteria propagated in experimentally infected rabbits (Fraser et al., 1998; Matĕjková et al., 2008; Giacani et al., 2010, 2014; Pětrošová et al., 2012, 2013; Zobaníková et al., 2012; Tong et al., 2017). The research community uses culture-independent enrichment techniques prior to whole genome sequencing of TPA clinical samples due to the overwhelming levels of human DNA and very low amounts of TPA DNA (1000:1 ratio of human to TPA DNA) found in clinical samples. However, available enrichment techniques demonstrate low efficiency (e.g., Anti-treponemal antibody enrichment, ATAE) (Grillová et al., 2018b) or are based on sequence-specific protocols (e.g., DNA-capture microarray and “in solution” capture techniques) (Arora et al., 2016; Pinto et al., 2016; Knauf et al., 2018; Marks et al., 2018), thus preventing the recovery of unique sequences not present in the reference genomes.

Genetically, TPA can be divided into two separate groups – SS14-like and Nichols-like strains (Nechvátal et al., 2014; Arora et al., 2016). As revealed by molecular typing studies of TPA isolates, most of the examined patients were infected with SS14-like strains (94.1%) (Woznicová et al., 2007; Flasarová et al., 2012; Grillová et al., 2014, 2018c; Arora et al., 2016; Gallo Vaulet et al., 2017; Mikalová et al., 2017a; Pospíšilová et al., 2018). The reason for the predominance of one genetic group is widely discussed, but was not clarified yet (Arora et al., 2016; Šmajs et al., 2016).

In this study, we performed direct whole genome sequencing of 25 TPA clinical samples isolated from different geographical areas using methyl-directed enrichment prior to next generation sequencing (NGS) (Barnes et al., 2014). Using this approach, we obtained 11 complete genome sequences, which represents the vast majority (92%) of complete TPA genomes sequenced directly from clinical samples. The subsequent detailed comparative genomic analyses revealed unexpected variability among Nichols-like genomes driven by inter-clade and/or intra-strain recombination events, which were accumulated mainly in the genes encoding predicted outer membrane proteins. This discovery, beyond being relevant to the understanding of basic biology of treponemes, highlights the presence of different repertoires of alleles coding for potential virulence factors, which circulate in the current human population.

## Results

### Clinical Samples

We selected 25 TPA samples recently isolated from 24 patients diagnosed with syphilis for whole genome sequencing. The sample set was selected to contain (i) samples with the highest possible genetic diversity, (ii) samples from different geographical areas and (iii) samples representing contemporary TPA infections. The samples were collected in four countries on three different continents (Australia, Cuba, Czechia, and France), mostly from males (92%) from which 74% were MSM (Table 1). Samples were taken as genital (*n* = 16), anal (*n* = 4), buccal (*n* = 3), or skin smears (*n* = 1), with one sample of lung tissue from a fatal case of congenital syphilis. As a result of the non-random sample selection, Nichols-like strains were overrepresented in our sample set (31%) compared to their prevalence in the infected population (5.9%) (Šmajs et al., 2018). Samples belonged to 9 different sequencing types (STs) and most (72%) carried the A2058G mutations in both *rrn* operons leading to resistance to macrolide antibiotics (Table 1).

**TABLE 1:** Clinical characteristics of samples and their genotyping data.

**ID**	**Origin**	**Year of isolation**	**Material**	**Sexual orientation**	**Stage**	**Allelic profile (ST)**	**Genetic group**	**Macrolide resistance/sensitivity (mutation)**
CW30^*^	Czechia	2014	genital smear	MSM	Primary	1.3.1 (1)	SS14-like	sensitive
CW84	France	2015	genital smear	MSW	Primary	1.3.1 (1)	SS14-like	resistant (A2058G)
CW85	France	2016	genital smear	MSM	Primary	1.3.1 (1)	SS14-like	resistant (A2058G)
CW87	France	2016	bucal smear	Unknown	Primary	1.23.1 (35)	SS14-like	resistant (A2058G)
CW88	Czechia	2017	genital smear	Unknown	Primary	1.3.1 (1)	SS14-like	resistant (A2058G)
CW56	Cuba	2013	genital smear	MSM	Unknown	1.3.1 (1)	SS14-like	resistant (A2058G)
CW82	Cuba	2016	genital smear	MSM	Unknown	15.7.3 (37)	Nichols-like	sensitive
CW65	Australia	2014	anal smear	Unknown	Secondary	9.14.3 (47)	Nichols-like	sensitive
CW83	Cuba	2015	genital smear	MSM	Unknown	9.24.8 (38)	Nichols-like	sensitive
CW86	France	2013	genital smear	MSM	Secondary	9.20.3 (31)	Nichols-like	sensitive
CW59	France	2012	anal smear	MSM	Secondary	9.7.3 (26)	Nichols-like	sensitive
CW57	Cuba	2014	genital smear	MSM	Unknown	1.3.1 (1)	SS14-like	resistant (A2058G)
CW51	Cuba	2016	anal smear	MSM	Unknown	1.3.1 (1)	SS14-like	resistant (A2058G)
CW53	Cuba	2015	genital smear	MSM	Unknown	1.3.1 (1)	SS14-like	resistant (A2058G)
CW29	Czechia	2013	genital smear	MSW	Primary	1.3.1 (1)	SS14-like	resistant (A2058G)
CW45^∗∗^	Czechia	2013	genital smear	WSM	Secondary	1.26.1 (25)	SS14-like	resistant (A2058G)
CW89	Czechia	2017	genital smear	MSW	Primary	1.26.1 (25)	SS14-like	resistant (A2058G)
CW33	Czechia	2012	lung	Unknown	Congenital	1.1.8 (3)	SS14-like	sensitive
CW35	Czechia	2013	genital smear	MSM	Primary/Secondary	9.7.3 (26)	Nichols-like	resistant (A2058G)
CW31	Czechia	2013	bucal smear	WSM	Secondary	1.26.1 (25)	SS14-like	resistant (A2058G)
CW44^∗∗^	Czechia	2013	bucal smear	WSM	Secondary	1.26.1 (25)	SS14-like	resistant (A2058G)
CW52	Cuba	2016	genital smear	MSM	Unknown	1.3.1 (1)	SS14-like	resistant (A2058G)
CW55	Cuba	2015	skin smear	MSM	Unknown	1.3.1 (1)	SS14-like	resistant (A2058G)
CW58	France	2013	genital smear	unknown	Unknown	9.7.3 (26)	Nichols-like	resistant (A2058G)
CW61	Czechia	2014	anal smear	MSM	Primary	1.3.1 (1)	SS14-like	resistant (A2058G)

^*^Sample enriched by ATAE (Grillová et al., 2018b) prior to the *Dpn*I enrichment. ^∗∗^Parallel samples taken from the same patient but isolated from different clinical material. MSM, men who have sex with men; MSW, men who have sex with women; WSM, women who have sex with men.

The number of TPA DNA copies as well as human DNA copies were determined by qPCR in all examined samples. The number of TPA DNA varied from 1 to 10^5^ copies per μl with the TPA DNA/human DNA ratio ranging from 0.01 to 3.69 (Supplementary Table S1). Given the fact that the human genome is approximately 3000 times larger than the TPA genome, the samples contained 10^3^–10^5^ times more human DNA than treponemal DNA requiring TPA DNA enrichment prior to sequencing.

### Methyl-Directed Enrichment Using Restriction Endonuclease *Dpn*I

The method we are presenting in this paper is based on binding activity of the *Dpn*I endonuclease. *Dpn*I is a restriction endonuclease that recognizes DNA methylated on adenine residue within the GATC sequence. This methylated DNA motif occurs in all bacteria that have deoxyadenosine methyltransferase (DAM) and is not present in higher eukaryotes. Immobilized *Dpn*I proteins on magnetic beads were used for specific capture of prokaryotic DNA. In the absence of Mg^2+^ ions, *Dpn*I binds its recognition sequence without cutting it. A previous study by Stamm et al. (1997) identified methylated adenine residues in GATC sequences in the TPA genome. To ensure the appropriate binding of *Dpn*I and enrichment effectivity of the *Dpn*I enrichment, a pilot experiment on one sample (CW88; Table 1) was performed. Before enrichment, the sample was sequenced with 384,489,027 reads and 25,168 of them were mapped to the treponemal genome. This resulted in 92% broad genome coverage and median sequencing depth of 6.6× and more than 100 sequencing gaps. After enrichment, the same sample was sequenced with a similar number of reads (332, 564, 761) revealing 296, 978 treponemal reads, which resulted in a near complete genome (98%) with a median sequencing depth of 68×. Except for the paralogous and repetitive regions (listed in the Supplementary Table S2), which were excluded from the reference-guided approach, the draft genome of CW88 had even sequencing coverage depth without any detectable biases. This indicated that G^m6^ATC DNA motifs are evenly distributed across the TPA genomes and that the *Dpn*I method is suitable for the TPA DNA enrichment.

### Direct Whole Genome Sequencing

Methyl-directed enrichment was applied to all examined clinical samples (*n* = 25) prior to NGS (details in section “Materials and Methods”). We used the preliminary sequencing to calculate the appropriate number of reads needed for the best possible coverage in the next sequencing runs. The NGS statistics are given in the Supplementary Table S3. In order to determine if the new sequencing approach was comparable to the traditional pooled segment genome sequencing (PSGS) approach, we sequenced isolates from the same organism (strain Phi-1 and Grady) following each method and did not find any discrepancies between the genomes (data not shown). According to the preliminary sequencing results, we selected samples containing treponemal DNA showing the highest breadth coverage (>97%, *n* = 11) as candidates for complete genome sequencing and samples with lower coverage (69–97%, *n* = 8) as candidates for genome-wide analyses (with a depth of coverage of 3 or greater). The samples with the lowest coverage (<10%, *n* = 7) were excluded from further analyses (Supplementary Table S3). For the whole genome determination, we amplified and Sanger sequenced regions with low coverage (less than 3 good-quality reads; in average 10 regions per genome) as well as regions, which were excluded from the bioinformatic pipeline including paralogous regions (*tpr* genes and *rrn* operons) and repetitive regions (e.g., *arp* and TP0470 genes) (details in section “Materials and Methods”).

**FIGURE 1:**
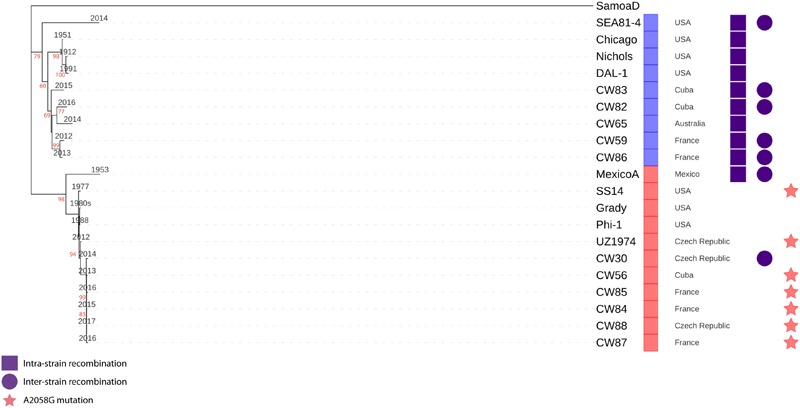
Phylogeny of all TPA complete genome sequences determined to date. Maximum likelihood bootstrapping method was used to generate the phylogenetic tree based on 2273 variable positions found exclusively in the whole genome sequences available to date (Table 2). The bootstrap values, when above 60, are given next to the branches in red. SS14-like strains are represented by red squares, Nichols-like strains by blue squares. The year of isolation is given next to the branches. Strains designated CW30-CW88 are the whole genome sequences determined in this study by *Dpn*I enrichment while Phi-1 and Grady were established in this study by PSGS. These represent 65% of all whole genome sequences of TPA to date. *tpr* genes, repetitive regions and inter- and intra-recombinant loci were excluded from this analysis (Supplementary Table S2 and Figures 2, 5). We used the genome of Samoa D (*T. pallidum* subsp. *pertenue*) as an outgroup.

**FIGURE 2:**
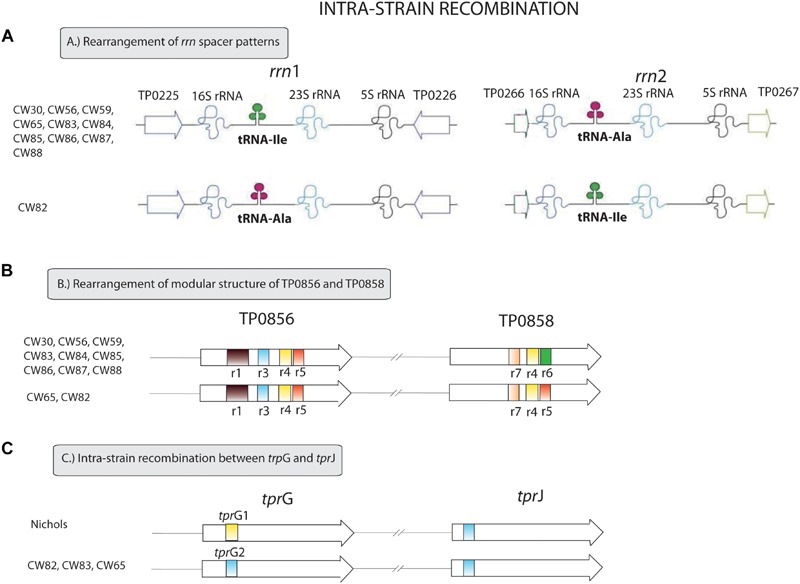
Intra-strain recombination events found among examined clinical samples. **(A)** Rearrangement of *rrn* spacer patterns. The ribosomal operon with Ile/Ala tRNA patterns differ from the ribosomal operon with Ala/Ile tRNA patterns in 34 nucleotide positions. The figure was modified according to Čejková et al. (2013). **(B)** Rearrangement of the modular structure of TP0856 and TP0858 genes. We identified two variants of the modular structure of the TP0856 and TP0858 genes – r1r3r4r5/r7r4r5 and r1r3r4r5/r7r4r6. These structures differ in 30 nucleotide sites. The figure was modified according to Strouhal et al. (2018). **(C)** Intra-strain recombination between *tpr*G and *tpr*J. *tpr*G1 and *tpr*G2 allele variants differ in 18 variable positions.

**FIGURE 3:**
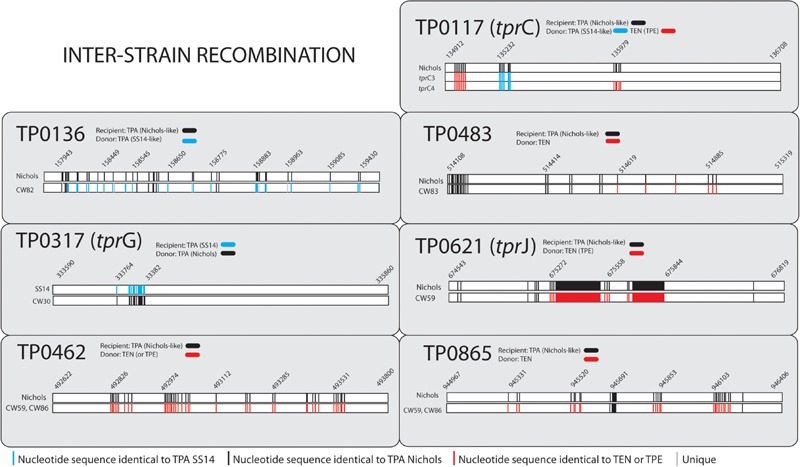
Inter-clade recombination events found among examined clinical samples. Coordinates according to the reference Nichols (CP04010.1) and reference SS14 (CP004011.2) for the Nichols-like and SS14-like clinical samples, respectively. Only positions with nucleotide differences are shown. The detailed description of the Figure 3 is given in the Supplementary Text S2.

All samples from SS14-like and Nichols-like clades revealed complete gene synteny. However, we identified significant differences between the genetics of these two TPA clades (Figure 1 and Supplementary Figure S1). The variability observed in Nichols-like strains was about one order of magnitude higher compared to that of the SS14-like strains. Moreover, in contrast to SS14-like strains, Nichols-like strains showed accumulation of a high number of single nucleotide variants (SNVs) in several genes. The manual inspection of the genes with a high SNV density (defined as more than 4 SNVs per gene) revealed that the genetic diversity was a result of inter-clade and/or intra-strain recombination events (altogether covering 147 SNVs, which represents 49.5% of variability found within Nichols-like strains). The intra-strain recombination events included rearrangements of genes coding for lipoproteins (TP0856 and TP0858), which possessed modular structures (Strouhal et al., 2018), *rrn* spacers and predicted virulence factors *tpr*G and *tpr*J (described below, Figure 2). The inter-clade recombination events were identified as sequences resembling both different syphilis genetic groups (i.e., SS14-like and Nichols-like groups) in the TP0136 gene encoding an outer membrane protein. In addition, the inter-clade recombination included sequences resembling both syphilis and bejel sequences (in TP0117 coding for TprC; in TP0317 coding for TprG; in TP0462 encoding probable lipoprotein; in TP0483 coding for hypothetical protein; in TP0621 coding for TprJ; in TP0865 encoding a putative outer membrane protein) (Table 2 and Figure 3).

**TABLE 2:** Inter-clade and intra-strain recombination in examined samples and in previously determined whole genome sequences of TPA.

			**Intra-strain recombination**				**Inter-clade recombination**								
	**Strain**	**Year of isolation**	***rrn*1/*rrn*2^1^**	**TP0858^2^**	***tpr*C/D^3^**	***tpr*G^4^**	**TP0136^5^**	**TP0326^6^**	**TP0462^7^**	**TP0483^8^**	**TP0488^9^**	**TP0865^10^**	***tpr*C^11^**	***tpr*G^12^**	***tpr*J^13^**
Nichols-like	Nichols (CP004010.2)	1912	Ile/Ala	R7R4R6	D	G1	–	–	–	–	–	–	C	–	–
strains	Chicago (CP001752.1)	1951	Ile/Ala	R7R4R6	D	G1	–	–	–	–	–	–	C	–	–
previously	DAL-1 (CP003115.1)	1991	Ile/Ala	R7R4R6	D	G1	–	–	–	–	–	–	C	–	–
described	SEA81-4 (CP003679.1)	2014	?	R3R4R5	D2	G2	–	–	–	–	–	Nichols/TEN	C	–	Nichols/TEN or TPE
Nichols-like	CW82 (CP34972)	2016	Ala/Ile	R7R4R5	D2	G2	Nichols/SS14	–	–	–	–	–	C3	–	–
strains in this	CW65 (CP34918)	2014	Ile/Ala	R7R4R5	D2	G2	–	–	–	–	–	–	C4	–	–
study	CW83 (CP34917)	2015	Ile/Ala	R7R4R6	D2	G2	–	–	–	Nichols/TEN	–	–	C3	–	–
	CW59 (CP34919)	2012	Ile/Ala	R7R4R6	D2	G2	–	–	Nichols/TEN or TPE	–	–	Nichols/TEN	C3	–	Nichols/TEN or TPE
	CW86 (CP34914)	2013	Ile/Ala	R7R4R6	D2	G2	–	–	Nichols/TEN or TPE	–	–	Nichols/TEN	C3	–	–
SS14-like	SS14 (CP004011.1)	1977	Ile/Ala	R7R4R6	D2	G1	–	–	–	–	–	–	C2	–	–
strains	Mexico A (CP003064.1)	1953	Ala/Ile	R7R4R6	D2	G1	–	SS14/TEN	–	–	SS14/TEN	–	C2	–	–
previously	Phi-1 (CP035193)	1988	Ile/Ala	R7R4R6	D2	G1	–	–	–	–	–	–	C2	–	–
described	Grady (CP035104)	1980s	Ile/Ala	R7R4R6	D2	G1	–	–	–	–	–	–	C2	–	–
	UZ1974 (CP028438.1)	2012	Ile/Ala	R7R4R6	D2	G1	–	–	–	–	–	–	C2	–	–
SS14-like	CW30 (CP034921)	2014	Ile/Ala	R7R4R6	D2	–	–	–	–	–	–	–	C2	SS14/Nichols	–
strains in this	CW84 (CP34916)	2015	Ile/Ala	R7R4R6	D2	G1	–	–	–	–	–	–	C2	–	–
study	CW85 (CP34915)	2016	Ile/Ala	R7R4R6	D2	G1	–	–	–	–	–	–	C2	–	–
	CW87 (CP34913)	2016	Ile/Ala	R7R4R6	D2	G1	–	–	–	–	–	–	C2	–	–
	CW88 (CP34912)	2017	Ile/Ala	R7R4R6	D2	G1	–	–	–	–	–	–	C2	–	–
	CW56 (CP34920)	2013	Ile/Ala	R7R4R6	D2	G1	–	–	–	–	–	–	C2	–	–

**FIGURE 4:**
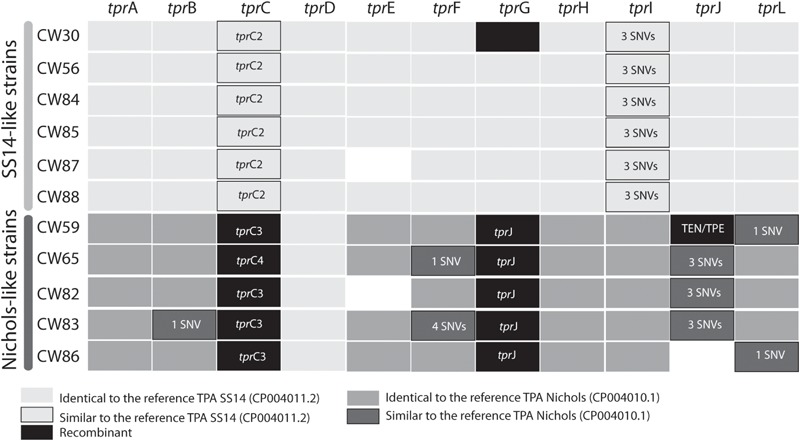
Allele variants of *tpr* genes found among examined clinical samples. To overcome the paralogous character of *tpr* genes, these loci were PCR amplified using primers with unique binding sites upstream and downstream the genes. The details are given in the section “Materials and Methods”. The allele variants are presented as a different color code (see legend under the figure). The blank spaces (e.g., *tpr*E in CW87 and CW82; *tpr*J in CW86) represent genome regions which were not amplifiable in the given samples. TprK variants are missing due to their hyper-variable sequences based on different subpopulations of the same strain. The *tpr*C allelic variants are shown in Supplementary Figure S2.

*^1^Intra-genomic rearrangements of *rrn* spacer patterns (Ile/Ala) (Čejková et al., 2013; Figure 3). ^2^Intra-genomic rearrangements of modular structure of TP0856 and TP0858 (Strouhal et al., 2018; Figure 3). ^3^Intra-genomic recombination of *tpr*C and *tpr*D. Also described in Kumar et al. (2018). ^4^Intra-genomic recombination of *tpr*G and *tpr*J (Figure 3). ^5^The inter-clade recombination of the TP0136 gene (Figure 4), reported also in Grillová et al. (2018c) and predicted in Arora et al. (2016). ^6^The inter-clade recombination of the TP0326 gene, as described in Pětrošová et al. (2012) and predicted in Arora et al. (2016). ^*7,8*^The inter-clade recombination of the TP0462 and TP0483 genes (Figure 4). ^9^The inter-clade recombination of the TP0488 gene, as described in Pětrošová et al. (2012). ^10–13^The inter-clade recombination of the TP0865, as predicted in Arora et al. (2016), *tpr*C, *tpr*G, and *tpr*J genes (Figure 4). Inter-clade and intra-strain recombination events are highlighted in gray.*

The detailed overview concerning different genome dynamics of SS14-like and Nichols-like strains are described in the Supplementary Text S1.

### Analyses of *tpr* Genes

When analyzing *tpr* genes among the SS14-like samples we did not find any significant variability with exception of the sample CW30, where we found a Nichols-like allele in the *tpr*G gene (differing in 29 SNVs from the SS14 sequence) probably as the result of an inter-clade recombination event (Figure 3). Otherwise, the SS14-like samples differed only in a few SNVs in the *tpr*C and *tpr*I genes compared to the SS14 reference (1 and 3, respectively, Figure 4 and Supplementary Figure S2).

In Nichols-like samples, the analyses of *tpr* genes revealed higher sequence variability compared to the group of SS14-like samples. We have identified gene conversion of a partial sequence of the *tpr*J gene into the *tpr*G gene (intra-strain recombination, Figure 2). Moreover, in sample CW59, we identified inter-clade recombination in the *tpr*J allele (with a putative TEN – *T. pallidum* subsp. *endemicum* or TPE – *T. pallidum* subsp. *pertenue* donor sequence, Figure 3). And finally, we have identified new alleles in the *tpr*C locus (*tpr*C3, *tpr*C4, Figure 3) represented by different branches in maximum likelihood phylogeny (Supplementary Figure S2). After manual inspection of the *tpr*C3 and *tpr*C4 alleles, we have identified the putative donor sequences of these alleles as SS14-like, bejel (TEN), or yaws (TPE) strains. The remaining *tpr* genes among Nichols-like strains were quite uniform, except for a few SNVs found in alleles present in the *tpr*B, *tpr*I, *tpr*J, *tpr*L loci (Figure 4).

### Recombination

When analyzing all published complete TPA genomes (including genomes from this study), we observed inter-clade or intra-strain recombination events frequently among Nichols-like strains, and only sporadically among SS14-like strains (Figure 1 and Table 2). More specifically, intra-strain recombination was found in all complete genomes of Nichols-like clade. Interestingly, inter-clade recombination was found more frequently in Nichols-like strains that were recently isolated (2013–2016) (Figure 1). To determine if the Nichols-like strains show differences in putative genes involved in recombination, we analyzed the *rec* genes (*rec*A, *rec*B, *rec*F, *rec*G, *rec*J, *rec*N, *rec*O, *rec*Q, *rec*R, *rec*X) and genes encoding recombinases (XerD1 and XerD2). Except for one SNV in the *rec*B gene distinguishing Nichols-like and SS14-like strains (and encoding non-synonymous amino acid substitution), no other differences were found.

### Analyses of the Strains Based on the Conserved Genomic Regions

**FIGURE 5:**
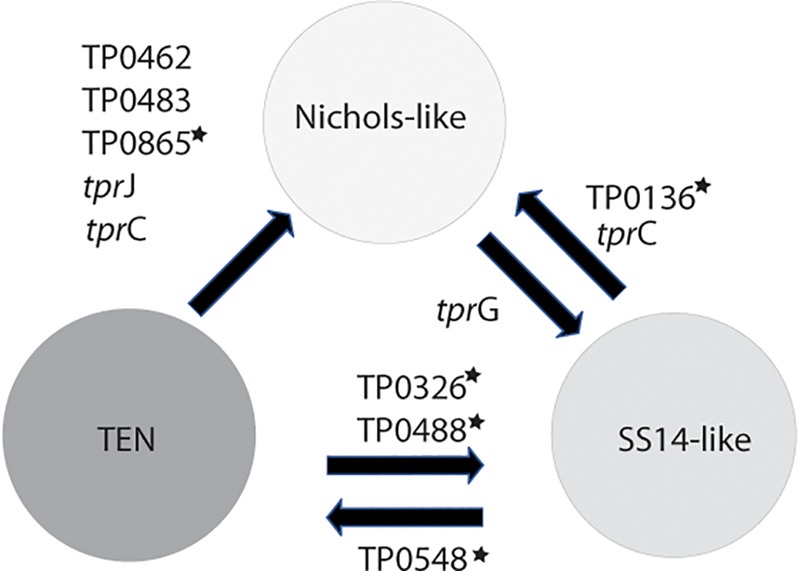
Inter-clade recombinations identified to date. The genes with stars represent loci, which were previously identified as recombinant (Pětrošová et al., 2012; Arora et al., 2016; Mikalová et al., 2017b; Grillová et al., 2018c). Five (out of 10) loci were identified in this study and were found among contemporary clinical samples enriched by a sequence-independent enrichment method.

To analyze the phylogeny of TPA strains, we excluded all identified inter-clade and intra-strain recombinant loci identified in this study and in the previous studies (Figure 5) and variable genes such as *tpr* genes, and reconstructed Network phylogenetic tree of these two clades based solely on the conserved genomic regions. Except for Mexico A strain, SS14-like strains created a star-like topology as described previously (Arora et al., 2016) and the highest genetic distance (18 SNVs) was found between sample CW87 and the SS14 reference genome (Supplementary Figure S3). Nichols-like strains were found to be more genetically diverse than SS14-like strains with the minimum genetic distance represented by 12 SNVs in the case of DAL-1 (1991) and Nichols (1912), and the highest genetic distance represented by 160 SNVs in the case of SEA81-4 (2014) and DAL-1 (1991).

Interestingly, SNVs distinguishing Nichols-like and SS14-like strains code for a significantly higher proportion of synonymous substitutions of amino acids (45.3%) compared to the SNVs found inside the SS14-like strains (17%) and within the Nichols-like strains (18%), suggesting that separate evolutionary forces operate inside and between each clade. Since we observed the same recombination events in the different phylogenetic branches (e.g., inter-clade recombination of TP0865 and *tpr*J in SEA-81-4 and CW59; Table 2 and Figure 1), these recombination events may have emerged several times independently.

### Recombination Loci Encode Putative Surface-Exposed Antigens

**FIGURE 6:**
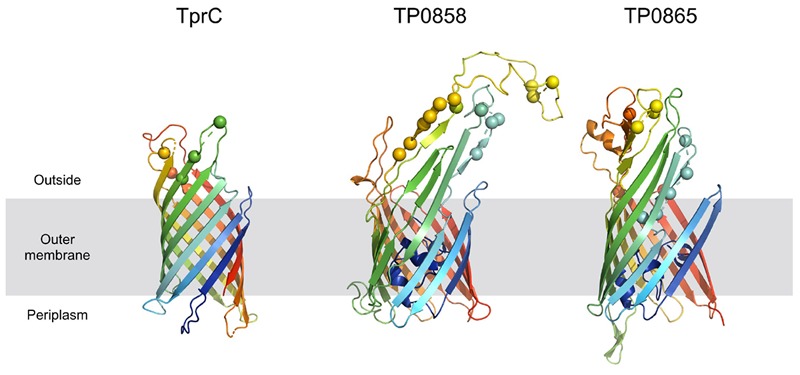
Homology models of TprC, TP0858, and TP0865. Homology models are shown in cartoon representation. The mutated residues are depicted with spheres. The models are colored based on a rainbow coloring scheme (with N-terminal of protein colored blue and C-terminal colored red).

Predicted protein structures encoded by recombinant loci provide important insights into structural and functional implications of recombination-driven variability. From the 8 recombinant protein-coding regions identified in this study, 6 were predicted to code for outer membrane proteins (TP0136, TP0858, TP0865, *tpr*C, *tpr*G and *tpr*J) and all of them contained predicted antigenic peptides (15–29). The prediction of treponemal protein structures is quite limited due to the lack of the protein homologues. However, a protein structure for *tpr*C was predicted by Centurion-Lara et al. (2013) and more recently by Kumar et al. (2018). The recombinant regions identified in the new *tpr*C3 and *tpr*C4 alleles found in this study (Supplementary Figure S2) correspond to the identified extracellular loops (L3, L4, L5) of the β-barrel outer membrane protein which were predicted to serve as B-cells epitopes^2^ (Kumar et al., 2018; Figure 6). Moreover, the newly predicted 3D structures of proteins TP0858 and TP0865 (Figure 6) revealed that the observed recombination-driven diversity almost strictly corresponds to the residues located at the host-pathogen interface.

## Discussion

Efforts to understand the pathogenesis of TPA have been hindered by the inability to routinely propagate the bacterium *in vitro* and the lack of an efficient method for obtaining genomes directly from clinical samples. TPA isolates form two separate clusters, i.e., SS14-like and Nichols-like clades (Pětrošová et al., 2013; Nechvátal et al., 2014; Arora et al., 2016; Šmajs et al., 2016, 2018; Figure 1 and Supplementary Figure S1). We have found striking genetic diversity of the contemporary Nichols-like strains when compared to SS14-like strains. Nichols-like strains represent only a minority (about 6%) of contemporary strains circulating in the syphilis-infected population (Woznicová et al., 2007; Flasarová et al., 2012; Grillová et al., 2014, 2018c; Arora et al., 2016; Gallo Vaulet et al., 2017; Mikalová et al., 2017a; Pospíšilová et al., 2018), as revealed by molecular typing studies of TPA isolates. Although there are several possible explanations available, the ultimate reasons for this contemporary worldwide predominance of SS14-like isolates in the human population remain unknown (Šmajs et al., 2016). However, the high prevalence of SS14-like strains circulating in the contemporary syphilis-infected population could be due to the recent expansion of these strains. Therefore, it would not be surprising that most of the SS14-like strains are in fact of more clonal character than the Nichols-like strains. The observed genetic diversity of the contemporary Nichols-like strains could, therefore, be a result of sampling bias. This possibility is supported by the existence of the sequentially diverse SS14-like strain, TPA Mexico A, isolated in 1953 (Pětrošová et al., 2012). To further address this question, additional molecular typing studies, accompanied by whole genome sequencing of genetically diverse MLST types, would be needed.

The methyl-directed enrichment used in this study allowed us to discover that about 50% of this genetic diversity was a result of inter-clade and/or intra-strain recombination events. Although molecular mechanisms of inter-clade and/or intra-strain recombination events could differ, both these processes can provide new alleles to TPA strains that are positively selected by the immune host system. In fact, most of the detected variability within both SS14- and Nichols-like clades predominantly led to non-synonymous amino acid changes which is consistent with positive selection of the corresponding genetic loci.

### Intra-Strain Recombinant Events

As described previously, the mechanisms resulting in intra-strain recombinant events include gene conversion in regions with modular character, e.g., *tpr* (*T. pallidum* repeat) genes (predicted to code for the potential virulence factors) (Gray et al., 2006; Strouhal et al., 2018), duplication or deletion of repetitive sequences (*arp*, TP0470) (Harper et al., 2008; Šmajs et al., 2018) and reciprocal translocation (*rrn* operons, *tpr*CD loci) (Čejková et al., 2013; Centurion-Lara et al., 2013).

The *tpr*D2 allele, but not *tpr*D (differing in 328 nucleotide positions), was previously predicted to be an outer membrane protein (Centurion-Lara et al., 2000), which suggests a different functional role for each allele during the course of infection. In our study, *tpr*D2 alleles were found among all completely sequenced TPA isolates (belonging to both SS14- and Nichols-like clades) despite the fact, that *tpr*D2 was previously believed to occur exclusively among SS14-like strains and *tpr*D allele among Nichols-like strains (Fraser et al., 1998; Zobaníková et al., 2012; Centurion-Lara et al., 2013). This suggests that in the ancestor of these Nichols-like strains, “*tpr*D allele” arose by duplication (gene conversion) of the *tpr*C gene. A similar finding was recently published by Kumar et al. (2018). Interestingly, although no such recombination was found among SS14-like strains, in the SS14 genome, a minor *tpr*D allele has been found in the *tpr*D locus (Pětrošová et al., 2013).

Similar recombination of predicted virulence factors identified among the clinical samples in this study was observed between the *tpr*G and *tpr*J genes resulting in a new *tpr*G2 allele in Nichols-like clinical samples. The same pattern of recombination was already predicted as possible in the work of Strouhal et al. (2018).

Another recombination found in this study resulted in new patterns in the TP0856 and TP0858 genes, following a pattern previously recognized among TPE strain Kampung-Dalan K363, TPA SEA81-4 (Strouhal et al., 2018) and TPE strains from the Solomon Islands (Marks et al., 2018). Both these proteins showed structural similarity to FadL, a long-chain fatty acid transporter (Kumar et al., 2018) required for the specific binding and transport of exogenous long-chain fatty acids prior to metabolic utilization. Moreover, the predicted 3D structures of TP0858 revealed that the recombination-driven diversity found almost entirely corresponds to residues located at the host-pathogen interface (Figure 6).

Finally, as a result of reciprocal translocation, we identified an inverse *rrn* spacer pattern in one of the Nichols-like samples. However, these *rrn* spacer patterns (Ile/Ala or Ala/Ile) appeared to be distributed randomly across species/subspecies classification, time and the geographical source of the treponemal strains (Čejková et al., 2013) and the impact of this intra-strain recombination remains unknown.

### Inter-Clade Recombinant Events

*Treponema pallidum* subsp. *pallidum* is not considered a competent bacterium and does not possess gene transfer mechanisms. In addition, no plasmid or phages have been described as of yet. Despite this, several apparent recombinant loci appear to result from inter-clade genetic recombinations. Previous studies identified such recombinations in TP0136 (Arora et al., 2016; Grillová et al., 2018c), TP0548 (Mikalová et al., 2017b), TP0326, TP0488 (Pětrošová et al., 2012), and TP0865 (Arora et al., 2016). In this study, we have identified five new recombinant loci representing one half of all inter-clade recombinant genes identified to date. We have identified recombination events between Nichols-like and SS14-like strains (in TP0117 [*tpr*C], TP0136, TP0317 [*tpr*G]); and Nichols-like strains and bejel treponemes (or possibly in some cases also TPE) (TP0117 [*tpr*C], TP0462, TP0483, TP0621 [*tpr*J], TP0865) (Figure 5). In the case of *tprG*, we have found both intra-strain recombinations (recombination between *tpr*G and *tpr*J genes resulting in a new *tpr*G2 allele) and inter-clade recombination (in the Nichols-like strain CW30 with sequence originating from SS14-like strains, Figure 3). In addition, the recombinant regions identified in the new *tpr*C3 and *tpr*C4 alleles found in this study (Supplementary Figure S2) correspond to the identified extracellular loops (L3, L4, L5) of the β-barrel outer membrane protein which were predicted to serve as B-cells epitopes (Kumar et al., 2018). Similarly, the newly predicted 3D structures of protein TP0865 in this study (Figure 6) showed the accumulation of recombination-driven diversity in the residues located at the host-pathogen interface.

While the intra-strain recombinant events are relatively easy to explain, the presence of inter-clade recombinations would require a DNA transfer to the recipient bacterium from the outside, likely during co-infection of patients with treponemes either belonging to two different syphilis clades (Nichols, SS14) or two different subspecies of *Treponema* (causing syphilis and bejel). Cross-immunity experiments (Turner and Hollander, 1957) revealed that there is no protective immunity between different TPA strains and between TPA and TEN strains enabling co-infections or overlapping infections of different treponemal strains. The subsequent homologous recombination of DNA taken up by recipient cells could provide alleles encoding protein sequences allowing persistence and escape from the immune response of the host.

In addition to inter-clade and intra-strain recombinations identified in this study, we have analyzed all publicly available TPA genome sequences (Supplementary Table S4) for the presence of such recombinant events. Among whole genome sequences (*n* = 20), we identified a SEA81-4 strain (Nichols-like strain; CP003679.1) that carries the *tpr*G2 allele and the r3r4r6 modular structure of TP0858 gene as results of intra-strain recombination events and we have identified TP0865 and *tpr*J as recombinant loci. Among draft genomes (*n* = 74), we have identified strains UW189B as inter-clade recombinant in both the TP0462 and TP0865 loci.

Given the adaptive evolution operating within both clades of syphilis treponemes, recombinant loci appear to be important in the treponemal pathogenesis and bacterium-host interactions. Despite the fact that TPA contains a low abundance of surface-exposed antigens, most of the Tpr proteins and recombinant proteins including TP0136, TP0326, TP0462, TP0483, TP0488, TP0548, and TP0865 encode for outer membrane proteins which are either targets for interactions with the immune system or structures enabling binding to host tissues (Brinkman et al., 2008; Arora et al., 2016; Kumar et al., 2018). In general, these proteins should be an important candidates for vaccine development. This is opportune, since several research teams are currently working on the development of a vaccine against syphilis. A comprehensive syphilis vaccine needs to react with antigens present in the reference strains but also on variants among contemporary TPA strains circulating in the human population. The current research identifying molecular types of TPA strains and their subsequent genomic analyses should be able to provide the required inventory of treponemal antigens and their variants.

## Materials and Methods

### Collection of Clinical Samples

Clinical samples were collected between 2013 and 2016 from several clinical departments in the Czechia (Department of Dermatovenerology, University Hospital Brno, Czechia); France (Institut Cochin U1016 Equipe Batteux, Laboratoire de Dermatologie–CNR Syphilis, Faculté de Médecine, Université Sorbonne Paris Descartes, Paris, France); Cuba (Instituto de Medicina Tropical “Pedro Kourí”, Havana, Cuba) and Australia (Melbourne Sexual Health Centre, Australia) (Table 1). Patients were considered as syphilis-positive when clinical symptoms were combined with positive syphilis serology or with positive PCR detection of treponemal DNA. All clinical samples were received after patients signed an informed-consent form and the written informed consent was obtained. The design of the study was approved by the ethics committee of the Faculty of Medicine, Masaryk University and the study was conducted in compliance with the Declaration of Helsinki.

### Isolation of DNA, MLST, and Quantification of Treponemal and Human DNA Present in the Clinical Material

Swab extracts (prepared by submersion of swabs into 1.5 ml of PBS and agitation for 5 min at room temperature) and tissue sample (25 mg) were used for isolation of DNA using a QIAamp DNA Blood Mini kit and a DNeasy Blood & Tissue Kit (QIAGEN, Hilden, Germany) according to manufacturer’s recommendations. Multi-locus sequence typing was performed as described previously (Grillová et al., 2018a). The sequences were submitted to BIGSdb of *T. pallidum* subsp. *pallidum* available at pubMLST (Grillová et al., 2019), and the allelic profiles, STs and clonal complexes were determined (Table 1).

The number of copies of treponemal and human DNA in samples subjected to the whole genome sequencing were determined by using Applied Biosystems^®^ 7500 Real-Time PCR System (United States). Treponemal DNA was detected by primers Tpa_polA_F (5′-GAGTGTGCAGTCCGCTATGC-3′) and Tpa_polA_R (5′-AGGCAAAAGCGGCATTTCTA-3′) amplifying 381 bp partial sequence of the *pol*A gene and the probe Tpa_polA_P (5′-FAM-TCCGCTTGGAAAGAGCA-BHQ1-3′) (Dubourg et al., 2015). Human DNA was detected by primers FP (5′-CCAAGTGTGAGGGCTGAAAAG-3′) and RP (5′-TGTTGTGGCTGATGAACTATAAAAGG-3′) targeting 80 bp partial sequence of the RNase P gene and the probe (5′-FAM-CCCCAGTCTCTGTCAGCACTCCCTTC-BHQ1-3′) (Chi et al., 2015). Real-time PCR mix was composed of 13 μl of QuantiFast^®^ Probe PCR Kit (QIAGEN, Hilden, Germany) with ROX dye passive reference, 0.1 μl of each primer and 0.05 μl of the probe of 100 μM (final concentration 0.4 and 0.2 μM, respectively). Total volume of each PCR reaction was 25 μl. Cycling conditions were 95°C (3 min); and 95°C (10 s), and 65°C (31 s) for 40 cycles.

### Methyl-Directed Enrichment Using Restriction Endonuclease DpnI

The endonuclease *Dpn*I cleaves the tetramer GATC when methylated at the N6 position of adenine. When used under conditions which prevent digestion, *Dpn*I binds the methylated tetramer which is distributed approximately every 256 bases on average in DAM positive bacteria such as TPA but is absent in mammalian genomes. This enables selective bacterial DNA enrichment from the excess of human DNA found in syphilis samples. To accomplish methyl-directed enrichment, clinical DNA samples (10–40 μl) were added to *Dpn*I-coated beads in 1.7 mL Eppendorf tubes in a final volume of 50 μl, as described previously (Barnes et al., 2014). The beads were mixed by end-over-end rotation for 30 min and the *Dpn*I-magnetic beads were separated using a magnetic stand. The beads were washed once with Wash Buffer (10 mM Tris pH 7.9, 500 mM NaCl, 10 mM CaCl_2_, 0.1% Tween 20) followed by a single Binding Buffer wash (10 mM Tris pH 7.9, 50 mM NaCl, 10 mM CaCl_2_, 0.01% Tween 20). DNA was eluted from beads by incubation with 20 μl of 5 M guanidinium thiocyanate at room temperature for 5 min and subsequently desalted via dialysis for 45 min using 20,000 MWCO Slide-A-Lyzer MINI dialysis cups (Thermo Scientific, Waltham, MA, United States).

### Next Generation Sequencing

The Nextera XT DNA library Preparation Kit (Illumina, San Diego, CA, United States) was used to produce barcoded libraries for all *Dpn*I enriched fractions. Library products were amplified using 19 cycles and DNA was purified with AMPure XP beads (New England BioLabs, Ipswich, MA, United States) with an elution volume of 20 μl. Library quality and size distributions were determined with the Lab Chip GX Touch-HT (Perkin Elmer, Waltham, MA, United States) and High Sensitivity DNA Analysis Kit (Perkin Elmer, Waltham, MA, United States). Libraries were diluted for sequencing and pooled as appropriate for the targeted sequencing depth on a single flow cell. NextSeq runs were prepared using NextSeq 500/550 300 Cycle High Output v2 (FC-404-2004), loaded at approximately 3.9 pM. All runs were configured to obtain 149 nucleotide paired-end read lengths.

The bioinformatic analysis was performed according to the pipeline described previously (Grillová et al., 2018b). Briefly, the quality check of the raw reads was performed using FastQC (v0.11.5, Andrews, 2010) and the raw reads were pre-processed using Cutadapt (v1.15, Martin, 2011) and Fastx-toolkit (v0.0.14, Gordon, 2014). The total set of pre-processed reads was mapped to the human genome reference (hg38) and the human-matching reads were removed using BBMap (v37.25, Bushnell, 2017). Subsequently, the remaining reads were mapped to the TPA reference genomes using BWA MEM (v0.7.15, Li, 2014). SS14-like strains were mapped to the SS14 TPA reference genome (GenBank Acc. No. CP004011.1) and Nichols-like strains were mapped to the Nichols TPA reference genome (GenBank Acc. No. CP004010.2). The post-processing of the mapping was performed using Samtools (v1.4, Li et al., 2009), Picard (v2.8.1, Broad Institute, 2015), GATK (v3.7, McKenna et al., 2010), and NGSUtils/bamutils (v0.5.9, commit a7f08f5, Breese and Liu, 2013). Low-quality mappings were omitted from the analyses (mapping quality; MAPQ < 40) as well as genome regions biased by mapping of short reads generated by NGS (e.g., repetitive and paralogous regions; Supplementary Table S2). Possible cross-mapping artifacts from other prokaryotes were removed by filtering of minimal alignment length (35 bp), maximum allowed mismatches (5 mismatches or 5% of the read length), and maximum soft-clipping (5% of the read length). The broad coverage was established based on at least three good-quality aligned reads. In parallel, *de novo* assemblies were performed by SPAdes assembler (v 3.11.1, Bankevich et al., 2012) using unfiltered reads aligned to the TPA reference (*k*-mer = 15, 21, 27, 33, 55, 77, 99, 127; minimal contig coverage 5). QUAST (v4.5, Gurevich et al., 2013) was used to evaluate the completeness of the assembly. Consensus sequences from reference-guided mapping and the *de novo* assemblies of TPA genomes were manually combined to create a final WGS for each sample. Jupyter Notebook containing workflow for methyl-directed enrichment sequencing analyses can be found at https://github.com/opplatek/bacterial_genome_analysis.

### SNV and Indel Identification

Single nucleotide variants and insertions and deletions (indels) were identified using FreeBayes (v0.9.21-19-gc003c1e, Garrison and Marth, 2012) and filtered by vcflib (v 0.1.15, Garrison, 2016). Prior to the variant call, mappings were indel realigned (GATK) and PCR duplicates were removed (v3.7, Broad Institute, 2015). FreeBayes was run with recommended settings for bacterial samples by the tool authors (ploidy 1; minimal coverage depth 3; minimal variant quality 50). Filtered variants were annotated using SnpEff (v4.2, Cingolani et al., 2012) with corresponding GenBank annotations.

### Completion of Whole Genomes

In the samples with the highest NGS broad coverage (>97%, *n* = 11), the Sanger sequencing of regions with low coverage was performed (approximately 10 regions for every genome). Moreover, paralogous *tpr* genes (*tpr*C, *tpr*D, *tpr*E, *tpr*F, *tpr*G, *tpr*I, *tpr*J) were amplified with Long-range PCR (Supplementary Table S2) under conditions described in the *Quality of treponemal DNA* paragraph and Sanger sequenced using sequencing primers presented in the Supplementary Table S5. The number of 60 bp-long repetitions in the *arp* gene and the number of 24 bp-long repetition in the TP0470 gene were determined by Sanger sequencing using primers listed in the Supplementary Table S2. The number of repetitions was verified by gel electrophoresis. To determine the intergenic spacers between rRNA encoding *rrn* loci, we designed primers for nested PCR using one unique primer in outer step for distinguishing Ala/Ile and Ile/Ala patterns (Supplementary Table S6). The Sanger sequencing reads were combined with the Illumina sequencing reads using Lasergene software (DNASTAR v. 7.1.0.; DNASTAR, Madison, WI, United States). The workflow to obtain the whole genome sequences of TPA is given in the Supplementary Figure S4.

Sequences of treponemal rRNA operons (*rrn1, rrn2*) were searched against the *de novo* assemblies (BLAST) (v2.2.31+, Camacho et al., 2009) and all the hits with more than 90% identity, and alignment length of 100 bp were extracted in fasta and bed format (Bedtools) (v2.27.0, Quinlan and Hall, 2010). We have used BLAST to discover all potential sequences of rRNA regions or their fragments. We manually inspected all the BLAST hits and selected those that most likely represented the real rRNA regions.

### Phylogenetic Analyses

Maximum likelihood phylogeny based on whole genomes was done using MEGA (v6.0, Tamura et al., 2011) using the Tamura-Nei model and 1000 bootstrap replications. The visualization of the phylogenetic tree was done using iTOL (v4, Letunic and Bork, 2007). Median-joining (MJ) networks were generated with Network version 4 (Bandelt et al., 1999).

### Detection of Recombination Events

Recombination patterns were identified by manual inspection of gene sequences that had a high number of SNVs (identified by SNV call) and displayed phylogeny incongruent with the one derived from whole genome sequences. The high number of SNVs was defined as the presence of at least 4 SNVs per gene, which is about 10-times higher number of polymorphic sites than expected between TPA clades (Pětrošová et al., 2013; Šmajs et al., 2016), and this threshold was calculated from the previously published recombination events found in other treponemal genomes (Pětrošová et al., 2012; Štaudová et al., 2014). The gene tree topology was tested against the tree topology derived from the whole genome sequences.

### Pooled Segment Genome Sequencing (PSGS) of TPA Phi-1 and Grady Strains

Philadelphia 1 (Phi-1) strain was isolated in Philadelphia, United States in 1988 (Harper et al., 2008) and Grady strain was isolated in Atlanta United States in 1980s. Both strains were provided by David L. Cox (Centers for Disease Control and Prevention, Atlanta, GA, United States) as a rabbit testicular tissue containing treponemal cells. Whole genomic DNA was amplified from rabbit testicular tissue using QIAGEN REPLI-g kit (QIAGEN, Hilden, Germany) according to manufacturer’s instructions. Amplified DNA served as a template for *T. pallidum* intervals (TP intervals) amplification during the PSGS phase as described previously (Weinstock et al., 2000; Čejková et al., 2012; Strouhal et al., 2017).

The amplified TP intervals (*n* = 279 and 272) of the Phi-1 and Grady samples, respectively, were sequenced using the Illumina platform (NextSeq 500) at CEITEC (Brno, Czechia). To separate paralogous regions, the amplified TP intervals were labeled with multiplex identifier adapters and sequenced as four different samples (Nextera^TM^ XT DNA Sample Preparation Kit, Illumina Inc., Madison, WI, United States). The sequencing reads were trimmed (Trimmomatic) (v0.32, Bolger et al., 2014), and low-quality bases were removed with a sliding window (window length of 4 nt; average quality of at least Phred 17). The sequencing reads shorter than 50 bp were omitted from the analyses. Reads were analyzed with respect to four distinct pools and were *de novo* assembled using SeqMan NGen software (v4.1.0, DNASTAR, Madison, WI, United States) as well as mapped to the TPA reference genome (GenBank Acc. No. CP004011.1).

### Annotation of Complete Genomes

For gene annotation, Geneious software (v5.6.5, Biomatters ApS, Aarhus, Denmark) was used. The *tpr*K gene showed intra-strain variability in all samples and the corresponding nucleotides positions were denoted as “N.” Raw data were deposited with NCBI under BioProject number: PRJNA508872 and the complete genome sequences can be found under the following Accession Numbers: CP034921, CP034920, CP034919, CP034918, CP034972, CP034917, CP034916, CP034915, CP034914, CP034913, CP034912, CP035104, and CP035193.

### 3D Structure Prediction

The 3D structures of TP0858 and TP0865 were generated using the SWISS-MODEL server (Waterhouse et al., 2018) using as templates the Protein Data Bank entries 3DWO and 3BS0, respectively. HHblits was used to find suitable template models (Remmert et al., 2011). The orientation of proteins with respect to the outer membrane corresponded to that predicted in the OPM database^1^ (Lomize et al., 2012). The TprC model was built using the TMBpro server (Randall et al., 2008) according to Kumar et al. (2018). The antigenic peptides were predicted by “Predicted Antigenic Peptides tool^2^.” Predictions were based on a table that reflects the occurrence of amino acid residues in experimentally known segmental epitopes. Segments were only reported if they had a minimum size of 8 residues.

## Data Availability

The datasets generated for this study can be found in NCBI, BioProject number: PRJNA508872.

## Ethics Statement

All patients signed the informed consent. The study protocol was approved by the Ethics Committee of all institutions involved in this study and was conducted in compliance with the Declaration of Helsinki.

## Author Contributions

LGr, RF, and DŠ designed the experiments. LGr, LM, MN, AN, PP, and CW performed the experiments. LGr, JO, AM, and DČ analyzed data. LGr, AAN, PG, ND, RS, MC, ID, and NA collected clinical samples. LGr, MP, and DŠ wrote the manuscript. All authors provided critical feedback.

## Conflict of Interest Statement

The authors declare that the research was conducted in the absence of any commercial or financial relationships that could be construed as a potential conflict of interest.

## Footnotes

1 https://opm.phar.umich.edu

2 http://imed.med.ucm.es/Tools/antigenic.pl
