# Supplementary material for: Directly Sequenced Genomes of Contemporary Strains of Syphilis Reveal Recombination-Driven Diversity in Genes Encoding Predicted Surface-Exposed Antigens
Source: Front Microbiol. 2019 Jul 31;10:1691. doi: 10.3389/fmicb.2019.01691 (PMC6685089; doi:10.3389/fmicb.2019.01691)
Supplement: Supplementary file 1 [file Data_Sheet_1.docx]

**Supplementary information**

**Supplementary text 1**

***Comparative genomics of six complete genomes of SS14-like strains (excluding tpr genes, other paralogous regions and repetitive regions)***

As observed in several previous studies^1-4^, our data clearly showed that TPA is divided into two genetically distinct groups (SS14-like and Nichols-like) (Figure 1 and 2). The SS14-like strains were less diverse compared to Nichols-like strains. When excluding paralogous *tpr* genes and repetitive regions, their diversity was restricted only to a minimal number of SNVs (41 in total) present in just 33 genes located across the genomes. Interestingly, most of the SNVs led to amino acid replacements (83%), occurring mostly in genes encoding outer membrane proteins. Multiple in frame mutations of the same coding regions were found only in 5 genes, with the highest SNVs density found in TP0548 (encoding putative membrane protein, a FadL homologue), followed by TP0324 (putative outer membrane protein), TP0133 (outer membrane protein^5^), TP0705 (bifunctional membrane carboxypeptidase/penicilin-binding protein) and TP0326 (BamA, outer membrane protein). When each of the SS14-like whole genome sequences was compared to the reference SS14 genome CP004011.2), a total number of differences varied from 11 to 18 SNVs. When the SS14-like whole genome sequences were compared to each other, differences varied from 5 to 7 SNVs. We did not find any mutations in the intergenic regions (IGR) in the SS14-like strains. No SNVs or genome rearrangements were found when we compared genomes isolated from the same patient, but different clinical materials (CW44 and CW45).

***Comparative genomics of five complete genomes of Nichols-like strains (excluding tpr genes, other paralogous regions, and repetitive regions)***

Nichols-like strains were more diverse with a higher number of SNVs accumulated in their genomes. Compared to the reference Nichols genome (CP004010.1), newly sequenced Nichols-like strains differed in 95-145 SNVs. These sequences differed in 20-72 SNVs between each other. The variability observed in Nichols-like strains (in total 350 SNVs) was about one order of magnitude higher compared to the SS14-like strains. Most of the SNVs found in Nichols-like clade led to amino acid replacements (n=271; 77.4%) as in the SS14 like clade. However, in the Nichols-like clade, we have found few SNVs in the inter-genic regions (3-5) and one SNV leading to the fusion of two neighboring genes (TP0006 and TP0007). Additionally, we identified 3 deletions (6-18 nt in length) and 5 insertions (1-12 nt) in the Nichols-like clade.

***Analyses of paralogous and repetitive regions***

The sequences of 5S, 16S, and 23S rRNA genes were identical among all examined samples, except for the presence of A2058G mutation in the 23S rRNA gene leading to the resistance to macrolide antibiotics (Table 1). Moreover, 5S, 16S, and 23S rRNA gene sequences were identical between both copies of these genes within one genome, respectively, including the presence of A2058G mutation. Most of the samples contained the same *rrn* spacer pattern (tRNA-Ile/tRNA-Ala), except of one sample (CW82, Nichols-like), where we observed the tRNA-Ala/tRNA-Ile pattern (Figure 3).

Interestingly, while 60-bp repeat region in the arp gene (TP0433) contained similar or slightly higher number of repetitions in the SS14-like strains (11-14) compared the Nichols-like strains (8-14), the number of 24-bp repetitions in the TP0470 gene was lower in SS14-like strains (4-15) compared to Nichols-like strains (14-23).

**Supplementary text 2**

There were 14 and 36-43 SNVs in the TP0117 gene (*tpr*C; 134912-136708; numbered according to CP004010.1) when comparing TPA Nichols (*trp*C), TPA SS14 (*tpr*C2) and TEN (TPE). *Tpr*C3 allele (found in Nichols-like strains) shared 37 nucleotide differences with TPA Nichols, 34 with TPA SS14 and 23 nucleotide differences with TEN/TPE. *Tpr*C4 allele (found in Nichols-like strain CW65) shared 32 nucleotide differences with TPA Nichols, 32 with TPA SS14 and 26/25 nucleotide differences with TEN/TPE. There were 49 SNVs in the TP0136 gene (157943-159430; numbered according to CP004010.1) when comparing TPA Nichols and TPA SS14. Sample CW82 (Nichols-like strain) shared 19 nucleotide differences with TPA Nichols (38.8%) and 30 nucleotide differences with TPA SS14 (61.2%). There were 29 SNVs in the TP0317 gene (*tpr*G; 333590-335860; numbered according to CP004011.1) when comparing TPA Nichols and TPA SS14. Sample CW30 (SS14-like strain) shared 29 nucleotide differences (100%) with TPA Nichols. There were 47 SNVs in the TP0462 gene (492622-493800; numbered according to CP004010.1) when comparing TPA Nichols and TEN Bosnia A. Samples CW59 and CW86 (Nichols-like strains) shared 8 nucleotide differences with TPA Nichols (17%) and 39 nucleotide differences with TEN Bosnia A (83%). There were 32 SNVs in the TP0483 gene (514108-515319; numbered according to CP004010.1) when comparing TPA Nichols and TEN Bosnia A. Sample CW83 (Nichols-like strain) shared 26 nucleotide differences with TPA Nichols (81.25%) and 6 nucleotide differences with TEN Bosnia A (18.75%). There were 206 SNVs in the TP0621 gene (*tpr*J; 674543-676819; numbered according to CP004010.1) when comparing TPA Nichols and TEN Bosnia A. Sample CW59 (Nichols-like strain) shared 6 nucleotide differences with TPA Nichols (3%) and 200 nucleotide differences with TEN Bosnia A (97%). There were 25 SNVs in the TP0865 gene (944967-946406; numbered according to CP004010.1) when comparing TPA Nichols and TEN Bosnia A. Samples CW59 and CW86 (Nichols-like strains) shared 2 nucleotide differences with TPA Nichols (8%) and 23 nucleotide differences with TEN Bosnia A (92%).


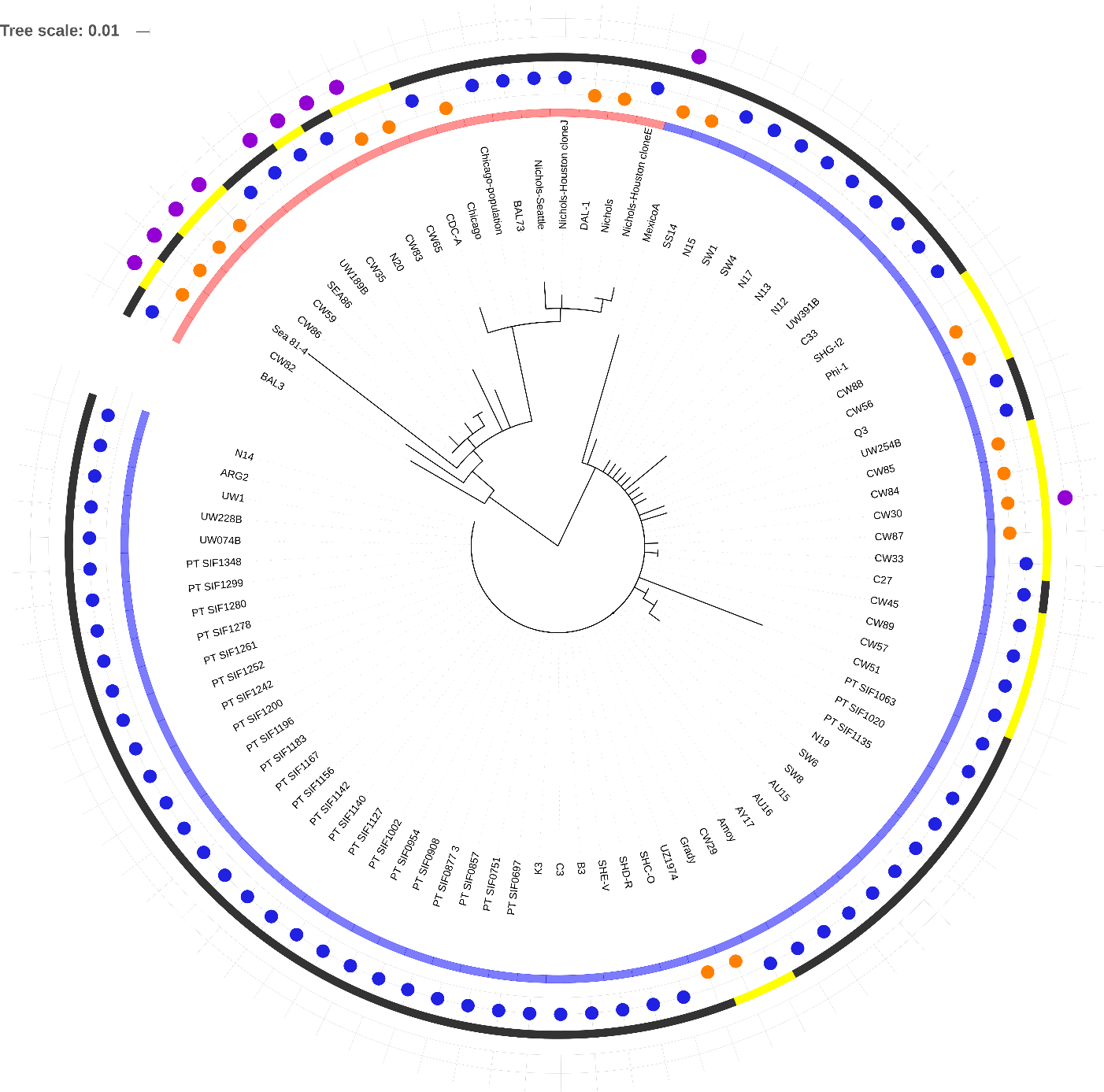


**Supplementary figure 1: Genome-wide phylogeny of all complete/draft genomes determined to date (n=92).** The detailed information about strains are given in the Table 1 and Supplementary Table 4. The maximum likelihood phylogeny is based on the 103 variable positions after removing the ambiguous regions as well as recombinant loci (Figure 3, Figure 7). Nichols-like strains are highlighted by red color and SS14-like strains with blue color. Complete genome sequences are marked with orange circles (n=20) and draft genomes with blue circles (n=72). The genomes determined in this study are marked with yellow color. Strains with identified inter-clade recombination events are marked with violet circles (this study,^2, 6, 7^).


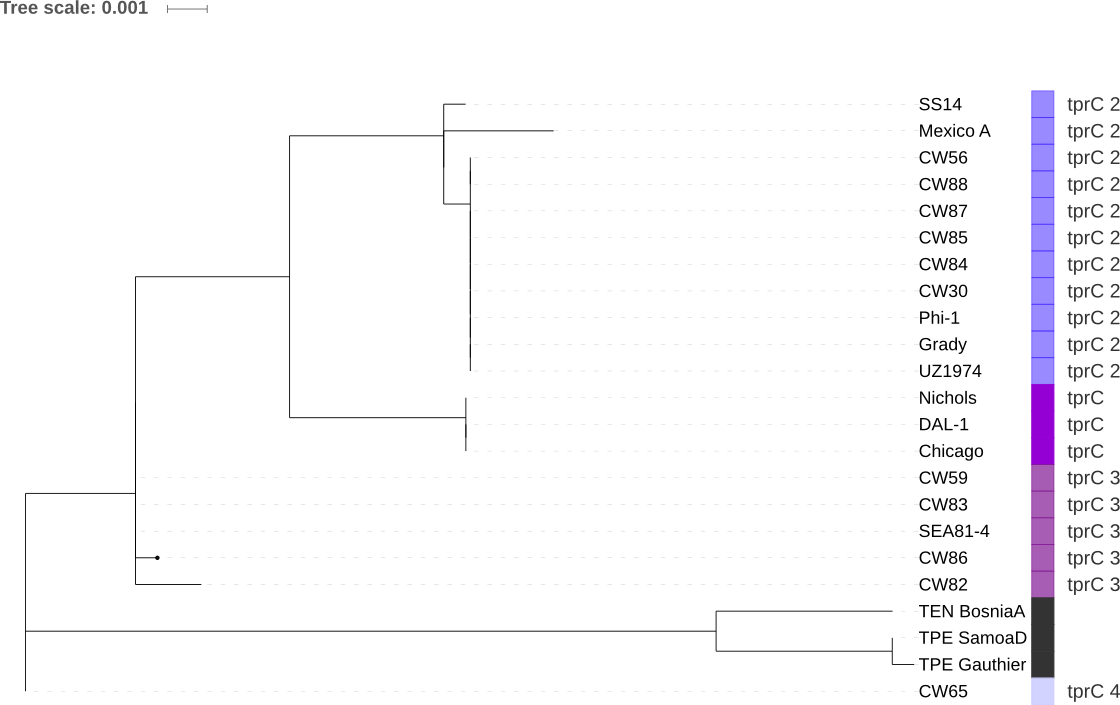


**Supplementary figure 2: Maximum likelihood phylogeny of the *tpr*C gene extracted from the whole genome sequences (Table 4)**. Several different alleles were found among the examined samples and were named *tpr*C2-*tpr*C4. The newly found alleles *tpr*C3 and *tpr*C4 are identical with the sequence of *tpr*C found among clinical samples in ^8^ (543 bp out of the total length of *tpr*C gene -1800 bp).


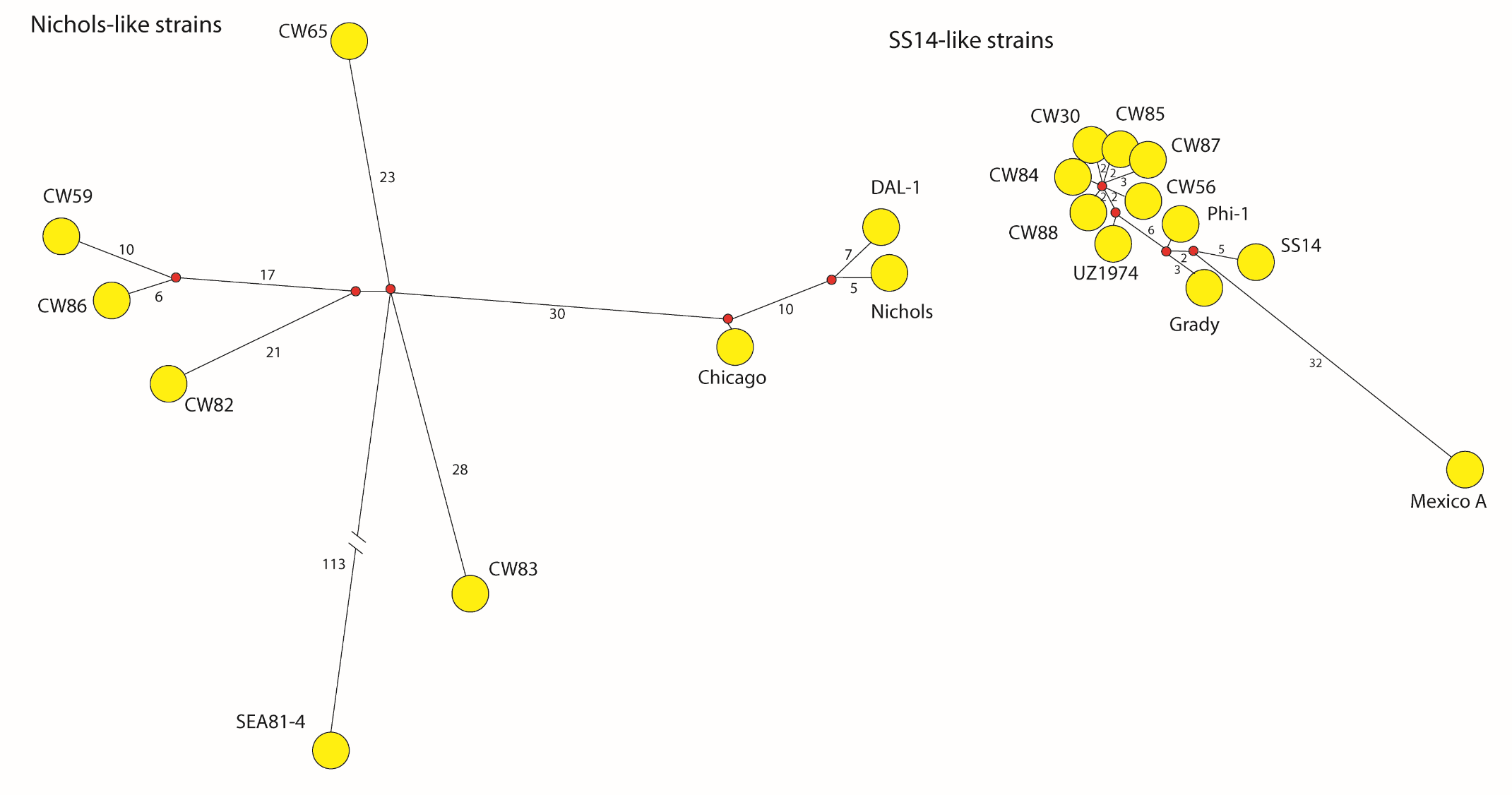


**Supplementary figure 3: Median-joining network of all available whole genome sequences of TP**. The phylotree was based only on the conserved genomic regions (excluding variable genes that arose due to the inter-clade or intra-strain recombination, *tpr* genes, repetitive regions and homo-polymeric regions). Every yellow circle represents a whole genome**.** The number of mutations, when > 1, is given close to branches. Median vectors are shown as small red connecting circles. If contiguous, indels were considered as a single event only.


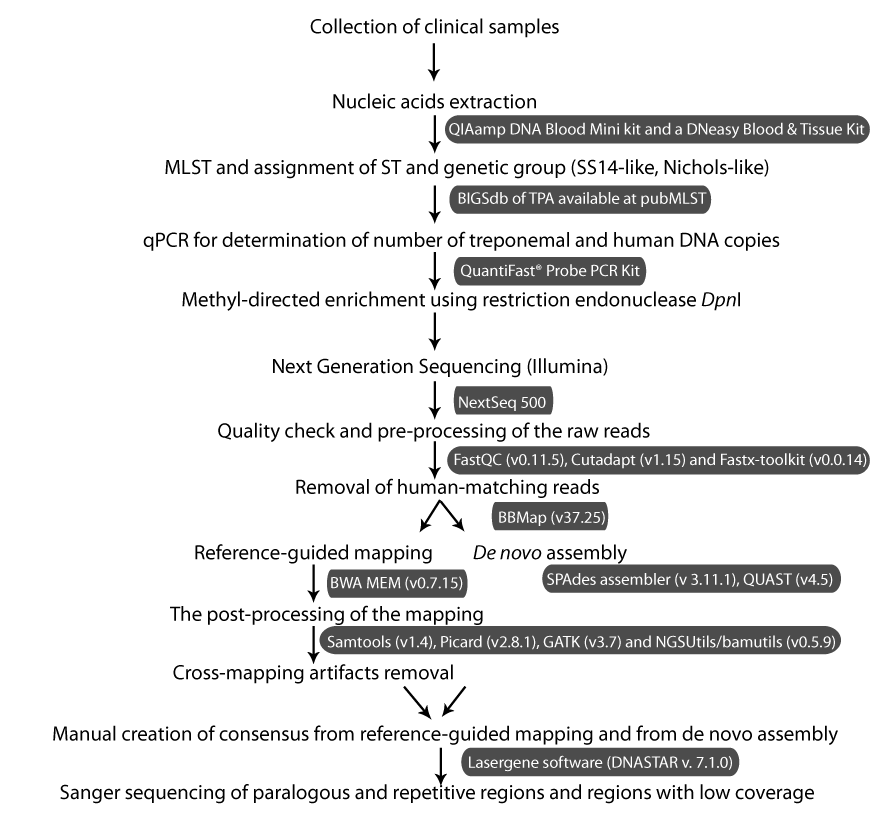


**Supplementary figure 4: Workflow chart for obtaining whole genome sequences of TPA.**

Supplementary Table 1: Quantification of TPA DNA, human DNA, quality control of the samples and percentage of TPA reads from the total number of bacterial reads

| ID | Number of TPA DNA copies/ µl | Number of human DNA copies/ µl | TPA DNA/human DNA ratio | Complete/draft genome | % of TPA reads from the total  number of bacterial reads |
| --- | --- | --- | --- | --- | --- |
| CW30 | 1.74 | 21.93 | 0.08 | Complete | 98 |
| CW84 | 13142.80 | 91501.03 | 0.14 | Complete | 69 |
| CW85 | 9887.34 | 2682.80 | 3.69 | Complete | 87 |
| CW87 | 1722.84 | 17328.88 | 0.1 | Complete | 1 |
| CW88 | 6881.88 | 13803.55 | 0.5 | Complete | 100 |
| CW56 | 14415.95 | 22961.58 | 0.63 | Complete | 87 |
| CW82 | 999.06 | 2981.17 | 0.34 | Complete | 57 |
| CW65 | 36568.61 | 30064.61 | 1.22 | Complete | 100 |
| CW83 | 296.59 | 245.04 | 1.21 | Complete | 73 |
| CW86 | 1460.26 | 2149.10 | 0.68 | Complete | 100 |
| CW59 | 46829.60 | 36490.92 | 1.28 | Complete | 48 |
| CW57 | 815.88 | 1217.13 | 0.67 | Draft | 40 |
| CW51 | 923.81 | 932.24 | 0.99 | Draft | 49 |
| CW53 | 459.07 | 169.56 | 2.71 | Draft | 40 |
| CW29 | 1786.09 | 3053.42 | 0.58 | Draft | 20 |
| CW45 | 2419.62 | 87518.47 | 0.03 | Draft | 1 |
| CW89 | 344.86 | 5238.56 | 0.07 | Draft | 100 |
| CW33 | 77.03 | 3676.51 | 0.02 | Draft | 100 |
| CW35 | 134.86 | 684.55 | 0.2 | Draft | 36 |
| CW31 | 1.00 | 60.12 | 0.02 | Draft | 1 |
| CW44 | 9.60 | 706.22 | 0.01 | Draft | 1 |
| CW52 | 1214.20 | 1514.79 | 0.8 | Draft | 46 |
| CW55 | 959.55 | 3409.15 | 0.28 | Draft | 100 |
| CW58 | 1553.08 | 25779.00 | 0.06 | Draft | 50 |
| CW61 | 2716.51 | 10253.38 | 0.26 | Draft | 66 |

Supplementary Table 2: Primers used for Long-range PCR for *tpr* gene amplification and primers used for determination of number of repetitions in *arp* and TP0470 genes.

| Locus | Primer ID | Sequence (5-3) | Coordinates* | Size of the PCR product (bp) |
| --- | --- | --- | --- | --- |
| *tpr*C | ES-42F | TTATCAGCCTGAATCGTATGTCCC | 133144-133167 | 4385 |
|  | TPI-11A-R | AGAGCCATTGTTGCAGTCTC | 137959-137978 |  |
| *tpr*D | TP0131-XL-F | AAGCTAAACGAGCCTCCACA | 150275-150294 | 3269 |
|  | cdc_TP0132-133Rseq | GCACACAGTGTTGCCCATAA | 153548-153567 |  |
| *tpr*E | ES-26F | TTCCGAGCCATATCTGCGTACT | 325002-325023 | 5764 |
|  | TPI-25AR | CGTTTAATGTTCTGCGGCCGGTGTTTG | 330739-330765 |  |
| *tpr*F | TPI-25(B)newF | GGGCGCCTTCCGGCAGGACTCT | 330289-330310 | 2001 |
|  | Nich323-R3 | TGTGCTGCTTCTGGTTATGC | 332270-332289 |  |
| *tpr*G | Nich323-F2 | GAAGACGCAAGCTCTACTGC | 332036-332055 | 3821 |
|  | ES-47R | GGAACCACTATCTCCTTCGAGACAA | 335832-335856 |  |
| *tpr*I | Bos3_TPESAMD_0619-bF | CACCCGAGTAGCACCACTTT | 670153-670172 | 3499 |
|  | TPI48-R15 | CTATACAGAACCTGCGTGCGTC | 673630-673651 |  |
| *tpr*J | 48-2F | GCACGGATGTATGCGGTGTAAG | 671288-671309 | 4017 |
|  | 48-2R | CTGGATACCTTGATTGGGCAGTAG | 675281-675304 |  |
| *arp* | TPI-32B-F7 | ACACTGCTGTTGAAATTAGC | 460732-461770 |  |
|  | TPI-32B-R10 | CGTAACGGTGGACAATGCTC | 461058-461538 | variable |
| TP0470 | 34-F3 | ACCGCTACAAAGAGGATAGG | 495206-499353 |  |
|  | 34-R3 | TCTACGCACAAAGAAAGAGC | 497142-497913 | variable |

*Coordinates according to TPA Nichols AE000520.1.

Supplementary Table 3: NGS statistics of the clinical samples.

| ID | No of total reads after QC | Number of TPA mapped reads | % of TPA mapped reads | Broad coverage 3/5* | Median depth |
| --- | --- | --- | --- | --- | --- |
| CW30** | 28506336 | 2509422 | 8.803032421 | 98,8/98,7 | 500 |
| CW84** | 378554269 | 954006 | 0.252013008 | 98,9/98.5 | 216 |
| CW85** | 377327639 | 1012407 | 0.268309791 | 98,9/98,5 | 221 |
| CW87** | 348118067 | 196551 | 0.056461017 | 98,2/97,5 | 42 |
| CW88** | 332564761 | 296978 | 0.089299299 | 98,2/98,1 | 68 |
| CW56** | 14899480 | 357796 | 2.401399243 | 98.8/98.6 | 84 |
| CW82** | 404889038 | 590033 | 0.145727087 | 98,6/98,3 | 127 |
| CW65** | 30645483 | 113875 | 0.371588204 | 97.1/96.3 | 27 |
| CW83** | 382892288 | 150290 | 0.039251248 | 97,7/97,3 | 35 |
| CW86** | 332913239 | 182529 | 0.054827799 | 97,9/97,3 | 38 |
| CW59** | 90920410 | 864304 | 0.950616039 | 98.6/98.5 | 199 |
| CW57*** | 100105253 | 63200 | 0.06313355 | 94,3/89,3 | 15 |
| CW51*** | 98172456 | 27233 | 0.02773996 | 69/53,1 | 6 |
| CW53*** | 108777757 | 61463 | 0.056503279 | 93,4/87,9 | 14 |
| CW29*** | 7217465 | 24288 | 0.336517046 | 77,9/57,4 | 6 |
| CW45*** | 150698270 | 57681 | 0.038275821 | 95.7/92.7 | 15 |
| CW89*** | 368983528 | 48826 | 0.013232569 | 94,4/87,3 | 11 |
| CW33*** | 159303021 | 39492 | 0.02479049 | 85.5/71.1 | 8 |
| CW35*** | 4717291 | 70066 | 1.485301628 | 96.8/86.2 | 17 |
| CW31**** | 193411760 | 385 | 0.000199057 | 0.6/0.1 | 1 |
| CW44**** | 10238662 | 1927 | 0.018820819 | 3,4/0,3 | 1 |
| CW52**** | 3604271 | 3390 | 0.094055081 | 9.4/1.7 | 1 |
| CW55**** | 294146 | 5544 | 1.884778307 | 19.7/4.2 | 2 |
| CW58**** | 247326 | 1756 | 0.709994097 | 2.4/ 0.1 | 1 |
| CW61**** | 162576 | 2409 | 1.481768527 | 4.6/0.4 | 1 |

*At least three good quality reads were used for establishment of the broad coverage.

**Samples with complete genomes.

***Samples with draft genomes.

****Samples with broad coverage lower than 20%, which were excluded from the analyses.

Supplementary Table 4: All available TPA genome sequences available in GenBank to date.

| Sample Name | Genetic group | Location | Year of isolation | Source | Genome (IDs) | References |
| --- | --- | --- | --- | --- | --- | --- |
| Nichols | Nichols-clade | USA | 1912 | rabbit inoculation | Complete (CP004010.2) | 9 |
| SS14 | SS14-clade | USA | 1977 | rabbit inoculation | Complete (CP004011.1) | 9 |
| Chicago | Nichols-clade | USA | 1951 | rabbit inoculation | Complete (CP001752.1) | 10 |
| Mexico A | SS14-clade | Mexico | 1953 | rabbit inoculation | Complete (CP003064.1) | 6 |
| DAL-1 | Nichols-clade | USA | 1991 | rabbit inoculation | Complete (CP003115.1) | 11 |
| SEA81-4 | Nichols-clade | USA | 1981 | rabbit inoculation | Complete (CP003679.1) | 12 |
| UZ1974 | SS14-clade | Czech Republic | 2012 | clinical acquired | Complete (CP028438.1) | 13 |
| Amoy | SS14-clade | China | 2011 | rabbit inoculation | Draft^1^ | 14 |
| ARG2 | SS14-clade | Argentina | 2013 | clinical acquired | Draft^1^ | 2 |
| AU15 | SS14-clade | Austria | 2013 | clinical acquired | Draft^1^ | 2 |
| AU16 | SS14-clade | Austria | 2013 | clinical acquired | Draft^1^ | 2 |
| AU17 | SS14-clade | Austria | 2013 | clinical acquired | Draft^1^ | 2 |
| C27 | SS14-clade | Czech Republic | 2012 | clinical acquired | Draft^1^ | 2 |
| C33 | SS14-clade | Czech Republic |  | clinical acquired | Draft^1^ | 2 |
| NE12 | SS14-clade | The Netherlands | 2013 | clinical acquired | Draft^1^ | 2 |
| NE13 | SS14-clade | The Netherlands |  | clinical acquired | Draft^1^ | 2 |
| NE14 | SS14-clade | The Netherlands |  | clinical acquired | Draft^1^ | 2 |
| NE15 | SS14-clade | The Netherlands | 2013 | clinical acquired | Draft^1^ | 2 |
| NE17 | SS14-clade | The Netherlands | 2013 | clinical acquired | Draft^1^ | 2 |
| NE19 | SS14-clade | The Netherlands | 2013 | clinical acquired | Draft^1^ | 2 |
| SW1 | SS14-clade | Switzerland | 2012 | clinical acquired | Draft^1^ | 2 |
| SW4 | SS14-clade | Switzerland | 2012 | clinical acquired | Draft^1^ | 2 |
| SW6 | SS14-clade | Switzerland | 2012 | clinical acquired | Draft^1^ | 2 |
| SW8 | SS14-clade | Switzerland | 2012 | clinical acquired | Draft^1^ | 2 |
| UW1 | SS14-clade | USA | 2004 | rabbit inoculation | Draft^1^ | 2 |
| BAL3 | Nichols-clade | USA | 1973 | rabbit inoculation | Draft^1^ | 2 |
| BAL73 | Nichols-clade | USA | 1973 | rabbit inoculation | Draft^1^ | 2 |
| NE20 | Nichols-clade | Netherlands | 2013 | clinical acquired | Draft^1^ | 2 |
| SEA86 | Nichols-clade | USA | 1986 | rabbit inoculation | Draft^1^ | 2 |
| SHC_O | SS14-clade | China | 2014 | rabbit inoculation | Draft^1^ | 15, 16 |
| SHD_R | SS14-clade | China | 2014 | rabbit inoculation | Draft^1^ | 15, 16 |
| SHE_V | SS14-clade | China | 2014 | rabbit inoculation | Draft^1^ | 15, 16 |
| SGF_12 | SS14-clade | China | 2014 | rabbit inoculation | Draft^1^ | 15, 16 |
| B3 | SS14-clade | China | 2015 | rabbit inoculation | Draft^1^ | 15, 16 |
| C3 | SS14-clade | China | 2015 | rabbit inoculation | Draft^1^ | 15, 16 |
| K3 | SS14-clade | China | 2015 | rabbit inoculation | Draft^1^ | 15, 16 |
| Q3 | SS14-clade | China | 2015 | rabbit inoculation | Draft^1^ | 15, 16 |
| PT_SIF1348 | SS14-clade | Portugal | 2014 | clinical acquired | Draft^1^ | 17 |
| PT_SIF1127 | SS14-clade | Portugal | 2013 | clinical acquired | Draft^1^ | 17 |
| PT_SIF1135 | SS14-clade | Portugal | 2013 | clinical acquired | Draft^1^ | 17 |
| PT_SIF1140 | SS14-clade | Portugal | 2013 | clinical acquired | Draft^1^ | 17 |
| PT_SIF1142 | SS14-clade | Portugal | 2013 | clinical acquired | Draft^1^ | 17 |
| PT_SIF1156 | SS14-clade | Portugal | 2013 | clinical acquired | Draft^1^ | 17 |
| PT_SIF1167 | SS14-clade | Portugal | 2013 | clinical acquired | Draft^1^ | 17 |
| PT_SIF1183 | SS14-clade | Portugal | 2013 | clinical acquired | Draft^1^ | 17 |
| PT_SIF1196 | SS14-clade | Portugal | 2013 | clinical acquired | Draft^1^ | 17 |
| PT_SIF1200 | SS14-clade | Portugal | 2013 | clinical acquired | Draft^1^ | 17 |
| PT_SIF1242 | SS14-clade | Portugal | 2014 | clinical acquired | Draft^1^ | 17 |
| PT_SIF1299 | SS14-clade | Portugal | 2014 | clinical acquired | Draft^1^ | 17 |
| PT_SIF1252 | SS14-clade | Portugal | 2014 | clinical acquired | Draft^1^ | 17 |
| PT_SIF1261 | SS14-clade | Portugal | 2014 | clinical acquired | Draft^1^ | 17 |
| PT_SIF1278 | SS14-clade | Portugal | 2014 | clinical acquired | Draft^1^ | 17 |
| PT_SIF1280 | SS14-clade | Portugal | 2014 | clinical acquired | Draft^1^ | 17 |
| PT_SIF0877_3 | SS14-clade | Portugal | 2010 | clinical acquired | Draft^1^ | 17 |
| PT_SIF0751 | SS14-clade | Portugal | 2009 | clinical acquired | Draft^1^ | 17 |
| PT_SIF0857 | SS14-clade | Portugal | 2010 | clinical acquired | Draft^1^ | 17 |
| PT_SIF0697 | SS14-clade | Portugal | 2009 | clinical acquired | Draft^1^ | 17 |
| PT_SIF0908 | SS14-clade | Portugal | 2010 | clinical acquired | Draft^1^ | 17 |
| PT_SIF0954 | SS14-clade | Portugal | 2010 | clinical acquired | Draft^1^ | 17 |
| PT_SIF1002 | SS14-clade | Portugal | 2011 | clinical acquired | Draft^1^ | 17 |
| PT_SIF1020 | SS14-clade | Portugal | 2011 | clinical acquired | Draft^1^ | 17 |
| PT_SIF1063 | SS14-clade | Portugal | 2013 | clinical acquired | Draft^1^ | 17 |
| Chicago-population | Nichols-clade | USA | 1951 | rabbit inoculation | Draft^1^ | Unpublished data |
| CDC-A | Nichols-clade | USA | 2013 | rabbit inoculation | Draft^1^ | Unpublished data |
| Nichols-Seattle | Nichols-clade | USA | 1912 | rabbit inoculation | Draft^1^ | Unpublished data |
| Nichols-Houston_cloneE | Nichols-clade | USA | 2015 | rabbit inoculation | Draft^1^ | Unpublished data |
| Nichols-Houston_cloneJ | Nichols-clade | USA | 2015 | rabbit inoculation | Draft^1^ | Unpublished data |
| UW074B | SS14-clade | USA | 2004 | rabbit inoculation | Draft^1^ | Unpublished data |
| UW189B | Nichols-clade | USA | 2004 | rabbit inoculation | Draft^1^ | Unpublished data |
| UW228B | SS14-clade | USA | 2004 | rabbit inoculation | Draft^1^ | Unpublished data |
| UW254B | SS14-clade | USA | 2004 | rabbit inoculation | Draft^1^ | Unpublished data |
| UW391B | SS14-clade | USA | 2006 | rabbit inoculation | Draft^1^ | Unpublished data |

Supplementary Table 5: Sequencing primers for *tpr* genes

| Locus | Primer ID | Sequence (5-3) | Coordinates* |
| --- | --- | --- | --- |
| tprC | cdc_TP0116-117Fseq | ACATGCTGCACGCAAAAA | 134728-134745 |
|  | 12R-5955 | ACCGAACATAGAGCAAGGAG | 135593-135612 |
|  | TP0131bF | GGAAGAGCGGAAGACATCAC | 135593-135589 |
|  | N+D_TP0117F | CTACTGCCAGGTCCTTCAGC | 136412-136431 |
| tprD | cdc_TP0130-132_Fseq | CATGGCATTGGTGAGAAAGA | 150964-150983 |
|  | tprD_151527-546F | GAGCAGGTGGGTGTAGGGGT | 151527-151546 |
|  | tprD-outerF | GTAACCAACACCAGAGTAAC | 152049-152068 |
|  | tprD-152298-317Fseq | GGTGTCAGTACTATCCCAGG | 152298-152317 |
| tprE | TPI25A-F1 | ATAGTACTGACCCCACGCAC | 328598-328617 |
|  | TPI25B-F7 | TGGTTCGAGGGTGAGTCGGC | 328787-328806 |
|  | TP0315-317XLF-Petra | TTAAACGCACCAATGGAACA | 329063-329082 |
|  | tprE-329504-523F | GGATCAACGCTGTGGTGCAG | 329504-329523 |
|  | tprE-330396-415R | CTACCGTTTCGTTTGGCATT | 330396-330415 |
| tprF | TPI25B-Nich-323-R3 | TGTGCTGCTTCTGGTTATGC | 332270-332289 |
|  | tprF-330931-949F | GCCGCGTGCGTCCCCGTAC | 330931-330949 |
|  | 12R-6515 | GGAAACAGCGGAGTAGATGT | 331790-331809 |
|  | tprEG_332073-93-F | GCTGTGCCCACACCCCTGAGC | 332073-332093 |
| tprG | cdc_TP0312-317Rseq | TTTTCACTGAGGGTCAGACG | 333164-333183 |
|  | TPI25A-R1 | CGCAGCTGTGCACAGTACTC | 332544-332563 |
|  | TPI25B-R13 | CGGTAGAGCTTGCCAGCAATG | 333946-333966 |
|  | TPI48-F23 | AGCCGGGCAAAGTTCGTCAG | 334215-334234 |
|  | TPI48-F16 | GCTCAAACCCCTTCACCACC | 333427-333446 |
| tprI | tprI-670879-898F | GGGGGAGAGAGAAACACGCG | 670879-670898 |
|  | tprI-672902-919R | GGGTGAGGTAGAAGTGAG | 672902-672919 |
|  | tprI-672061-080F | GCGTCGAGGGCGAAGGAGAA | 672061-672080 |
|  | Bos3-TPESAMD0619aR | ACCTATGTGTCGGCTGGACT | 671614-671633 |
|  | 12F-5609 | TGTAACGGATGTAGGTGAGG | 671423-671442 |
| tprJ | TPI25B-F8 | CATTCCACAGCAGTTTATCC | 674426-674445 |
|  | TPI48-R15 | CTATACAGAACCTGCGTGCGTC | 673630-673651 |
|  | tprJ-674522-541R | AGGACACCCTCTGTGCACGG | 674522-674541 |
|  | tprJ-673193-212F | CCGTAACGCTTGGCTTCACC | 673193-673212 |

Supplementary table 6: Primers for Ala/Ile and Ile/Ale intergenic spacers distinction

| rrn1 | | | | |
| --- | --- | --- | --- | --- |
|  | primer ID | sequences (5-3) | PCR product (bp) |  |
| outer | RNA1F | GTGTGTGAGTCTGGCAGGAA** | 2351 |  |
|  | TP0225-6cR | TTAGGGTGGGGTGTGAAGTT |  |  |
| inner | TP0225MF | GGGCATGGCATGGTGCCGTG | 260 |  |
|  | TP0225-6cR | TTAGGGTGGGGTGTGAAGTT |  |  |
| rrn2 | | | | |
| outer | RNA2F | ACAAGTGAGCGAAGCGTTTT** | 2334 |  |
|  | TP0255-6cR | TTAGGGTGGGGTGTGAAGTT |  |  |
| inner | TP0225MF | GGGCATGGCATGGTGCCGTG | 267 |  |
|  | TP0255-6cR | TTAGGGTGGGGTGTGAAGTT |  |  |

*According to the reference SS14 genome (Acc. No. CP004011.2).

**Unique primers.

**Supplementary references**

1. Nechvátal, L. *et al.* Syphilis-causing strains belong to separate SS14-like or Nichols-like groups as defined by multilocus analysis of 19 *Treponema pallidum* strains. *Int. J. Med. Microbiol.* **304**, 645–653 (2014).
2. Arora, N. *et al.* Origin of modern syphilis and emergence of a pandemic *Treponema pallidum* cluster. *Nat Microbiol* **2**, 16245 (2016).
3. Šmajs, D., Mikalova, L., Strouhal, M. & Grillova, L. Why Are There Two Genetically Distinct Syphilis-Causing Strains? *FIDT* **7**, (2016).
4. Šmajs, D., Strouhal, M. & Knauf, S. Genetics of human and animal uncultivable treponemal pathogens. *Infect. Genet. Evol.* **61**, 92–107 (2018).
5. Naqvi, A. A. T., Shahbaaz, M., Ahmad, F. & Hassan, M. I. Identification of functional candidates amongst hypothetical proteins of *Treponema pallidum* ssp. *pallidum*. *PLoS ONE* **10**, e0124177 (2015).
6. Pětrošová, H. *et al.* Whole genome sequence of *Treponema pallidum* ssp. *pallidum*, strain Mexico A, suggests recombination between yaws and syphilis strains. *PLoS Negl Trop Dis* **6**, e1832 (2012).
7. Grillová, L. *et al.* Multilocus sequence typing of *Treponema pallidum* subsp. *pallidum* in Cuba from 2012 to 2017. *J. Infect. Dis.* (2018). doi:10.1093/infdis/jiy604
8. Kumar, S. *et al.* Sequence Variation of Rare Outer Membrane Protein β-Barrel Domains in Clinical Strains Provides Insights into the Evolution of *Treponema pallidum* subsp. *pallidum*, the Syphilis Spirochete. *MBio* **9**, (2018).
9. Pětrošová, H. *et al.* Resequencing of *Treponema pallidum* ssp*. pallidum* strains Nichols and SS14: correction of sequencing errors resulted in increased separation of syphilis treponeme subclusters. *PLoS ONE* **8**, e74319 (2013).
10. Giacani, L. *et al.* Complete genome sequence and annotation of the *Treponema pallidum* subsp. *pallidum* Chicago strain. *J. Bacteriol.* **192**, 2645–2646 (2010).
11. Zobaníková, M. *et al.* Complete genome sequence of *Treponema pallidum* strain DAL-1. *Stand Genomic Sci* **7**, 12–21 (2012).
12. Giacani, L. *et al.* Complete Genome Sequence of the *Treponema pallidum* subsp. *pallidum* Sea81-4 Strain. *Genome Announc* **2**, (2014).
13. Grillová, L. *et al.* Sequencing of *Treponema pallidum* subsp. *pallidum* from isolate UZ1974 using Anti-Treponemal Antibodies Enrichment: First complete whole genome sequence obtained directly from human clinical material. *PLoS ONE* **13**, e0202619 (2018).
14. Tong, M.-L. *et al.* Whole genome sequence of the *Treponema pallidum* subsp. *pallidum* strain Amoy: An Asian isolate highly similar to SS14. *PLoS ONE* **12**, e0182768 (2017).
15. Sun *et al.* Tracing the origin of *Treponema pallidum* in China using next-generation sequencing. - PubMed - NCBI. Available at: https://www.ncbi.nlm.nih.gov/pubmed/27344187. (Accessed: 24th January 2019)
16. Strouhal, M. *et al.* Reanalysis of Chinese *Treponema pallidum* samples: all Chinese samples cluster with SS14-like group of syphilis-causing treponemes. *BMC Res Notes* **11**, 16 (2018).
17. Pinto, M. *et al.* Genome-scale analysis of the non-cultivable *Treponema pallidum* reveals extensive within-patient genetic variation. *Nat Microbiol* **2**, 16190 (2016).
